# Imaging Neurodegenerative Diseases: Mechanisms and Interventions

**DOI:** 10.1155/2014/419317

**Published:** 2014-12-21

**Authors:** Lijun Bai, Lin Ai, Mingzhou Ding, Yong He, Lixing Lao, Fanrong Liang

**Affiliations:** ^1^The Key Laboratory of Biomedical Information Engineering, Ministry of Education, Department of Biomedical Engineering, School of Life Science and Technology, Xi'an Jiaotong University, Xi'an 710049, China; ^2^Department of Nuclear Medicine, Beijing Tiantan Hospital, Capital Medical University, Beijing 100050, China; ^3^J.Crayton Pruitt Family Department of Biomedical Engineering, University of Florida, Gainesville, FL 32610, USA; ^4^State Key Laboratory of Cognitive Neuroscience and Learning and IDG/McGovern Institute for Brain Research, Beijing Normal University, Beijing 100875, China; ^5^School of Chinese Medicine, University of Hong Kong, 10 Sassoon Road, Pokfulam, Hong Kong; ^6^Center for Integrative Medicine, School of Medicine, University of Maryland, Baltimore, Baltimore, MD 21201, USA; ^7^Chengdu University of Traditional Chinese Medicine, Chengdu 610075, China

Neurodegenerative diseases as diverse as Alzheimer's disease, Parkinson's disease, amyotrophic lateral sclerosis, multiple sclerosis, stroke, and depression are thought to share a common pathogenesis mechanism—the aggregation and deposition of misfolded proteins, which leads to progressive central nervous system impairments. This special issue compiled 11 articles, most of which are novel and excellent investigations in this field.

Two modalities of neuroimaging techniques (PET and MRS) are leading to a greater understanding of quantification for the early and differential diagnosis of Alzheimer's disease. P. Daniela et al. conducted a meta-analysis and GRADE analysis reporting differences in the levels of sensitivity and specificity for the standard visual FDG PET scan or dichotomous readout based amyloid PET with respect to parametric or semiquantitative analysis. The review by Radoslaw Magierski and Tomasz Sobow summarized the main results obtained from the application of neuroimaging techniques in Dementia with Lewy bodies (DLB) cases, mainly focusing on proton magnetic resonance spectroscopy (1H-MRS). DLB and Parkinson's disease share clinical symptoms and neuropsychological profiles. Proton magnetic resonance spectroscopy (1H-MRS) provides a noninvasive method of assessing an* in vivo* biochemistry of brain tissue.

The altered brain mechanisms underlying potential progress state related with neurodegenerative disease are addressed in four articles. As a subtype of mild cognitive impairments (MCI), amnestic mild cognitive impairment (aMCI) most often leads to Alzheimer's disease. Z. Zhao and colleagues aimed to elucidate the altered resting brain in patients with aMCI. They found increased activities in the frontal lobe of aMCI patients, which might indicate effective recruitment of compensatory brain resources. Traumatic brain injury (TBI) is one of the most consistent candidates for initiating the molecular cascades that result in Alzheimer's disease. Y. Zhu et al. enrolled only “probable and symptomatic” TBI with no visible lesions by using conventional and SWI neuroimaging techniques, while DTI analysis indicated widespread declines in the fractional anisotropy (FA) of gray matter and white matter, particularly in the limbic-subcortical structures. A better understanding of the acute changes occurring following symptomatic TBI may increase our understanding of neuroplasticity and continuing degenerative change, which in turn, may facilitate advances in management and intervention. S. Zheng et al. evaluated relationship between degree of internal carotid artery (ICA) stenosis and frontal activations induced by working memory (WM) task using fMRI. They demonstrated that cognitive impairments in ICA stenosis were associated with frontal lobe dysfunctions. Furthermore, D. Rong et al. investigated the involvement and changes of the corticospinal tract (CST) in patients with medulla infarct during motor recovery. The degree of degeneration and spared peri-infarct CST compensation may reflect important motor recovery mechanism.

Two researches mainly focus on the pathophysiological changes and nuclear neuroimaging diagnostic work-up for amyotrophic lateral sclerosis (ALS) in assessing the evolution of the disease and/or the effectiveness of therapeutic action. A. Cistaro et al. offered a comprehensive overview of the different radiotracers for the assessment of the metabolism of glucose (FDG), the measurement of cerebral blood flow (CBF), or the evaluation of neurotransmitters, astrocytes, and microglia in clinically diffuse radiopharmaceuticals in ALS. Q. Zhang et al. investigated abnormal lateralization of brain gray matter (GM) in the ALS patients and focused on the relationship between GM abnormalities and side of disease onset in limb-onset patients. They found a negative relationship between regional atrophy and disease progression rate, indicating the possible correspondence between disease progression and cortical abnormality. Depression is a risk factor for neurodegenerative diseases in general, including Alzheimer's disease (AD). Its premorbid signs are commonly observed, and the morbidity of depression is higher in dementia patients. R. Yang and colleagues examined the effects of antidepressant treatment (sertraline) on hypothalamus-related resting brain networks. People with multiple sclerosis (MS) are also at high risk of depression. One study from Y. Shen et al. reported that there was a significant relation between the depression symptoms in relapsing-remitting MS and global microstructural changes both in brain white matter and gray matter. P. Zhang et al. demonstrated altered functional connectivity (FC) in the affective network (AN) in patients with poststroke depression.

By gathering these papers, we hope to enrich our readers and researchers with respect to the underlying neurological mechanism of neurodegenerative diseases. We look forward to an increasing number of both clinical trials and experimental studies to further identify early disease biomarkers and more effective therapies to improve the quality of life and cognitive function of the patients affected by these devastating illnesses.

*Lijun Bai* *Lin Ai* *Mingzhou Ding* *Yong He* *Lixing Lao* *Fanrong Liang*

